# Effect of Tempo on Temporal Expectation Driven by Rhythms in Dual-Task Performance

**DOI:** 10.3389/fpsyg.2021.755490

**Published:** 2021-12-08

**Authors:** Zhihan Xu, Yanna Ren, Yosuke Misaki, Qiong Wu, Sa Lu

**Affiliations:** ^1^Department of Foreign Language, Ningbo University of Technology, Ningbo, China; ^2^Department of Psychology, College of Humanities and Management, Guizhou University of Traditional Chinese Medicine, Guiyang, China; ^3^Laboratory of Cognitive Neuroscience, Graduate School of Natural Science and Technology, Okayama University, Okayama, Japan; ^4^School of Education, Suzhou University of Science and Technology, Suzhou, China

**Keywords:** temporal expectation, tempo, rhythm, dual-task, working memory

## Abstract

Temporal expectation is the ability to focus attention at a particular moment in time to optimize performance, which has been shown to be driven by regular rhythms. However, whether the rhythm-based temporal expectations rely upon automatic processing or require the involvement of controlled processing has not been clearly established. Furthermore, whether the mechanism is affected by tempo remains unknown. To investigate this research question, the present study used a dual-task procedure. In a single task, the participants were instructed to respond to a visual target preceded by a regular or an irregular visual rhythm under a fast (500 ms) or slow (3,500 ms) tempo. The dual-task simultaneously combined a working memory (WM) task. The results showed temporal expectation effects in which the participants responded faster to the regular than to the irregular conditions in a single task. Moreover, this effect persisted under dual-task interference in the fast tempo condition but was impaired in the slow tempo condition. These results revealed that rhythmic temporal expectation induced by fast tempo was dependent on automatic processing. However, compared with the faster tempo, temporal expectation driven by a slower tempo might involve more controlled processing.

## Introduction

Temporal expectations are critical to our survival because anticipating the moment of forthcoming events enables the brain to induce perception or action performance for the benefit of adaptive behavior. In many situations in our daily life, for instance, driving, walking, listening to music, or playing sports, the dynamics of the environment are generally non-random, and such environmental rhythm can be serviced to generate expectations and promote performance.

Empirical evidence showed that temporal expectations could be driven by isochronous sequences of stimuli (such as, rhythms). Numerous previous research studies have reported that individuals show speeded reaction time (RT) performance and improved perceptual discrimination in response to the rhythmically simple sequences compared with irregularly timed sequences (Barnes and Jones, 2000; Jones et al., 2002; Correa and Nobre, 2008; Lange, 2009, 2010; Rohenkohl and Nobre, 2011). These effects might be associated with entrainment, in which low-frequency neural oscillations in connection with fluctuations between periods of high and low neural sensitivity to the input and become phase-aligned to the external rhythmic stream such that periods of high sensitivity coincide with on-beat times of rhythmic stimuli (Jones, 1976; Large and Jones, 1999; Barnes and Jones, 2000; Schroeder and Lakatos, 2009; Breska and Deouell, 2016). A handful of studies failed to find the rhythmic behavioral benefits (Benwell et al., 2017; Ruzzoli et al., 2019; Vigué-Guix et al., 2020; Lin et al., 2021).

Temporal expectations driven by rhythms have been typically suggested due to a more automatic process, dissociated from the temporal expectation created by symbolic cues, which has been suggested to be created intentionally and voluntarily (Coull and Nobre, 2008; Trivino et al., 2011; de la Rosa et al., 2012; Breska and Deouell, 2014; Correa et al., 2014). However, it is a topic of debate whether the rhythm-based temporal expectation is only attributed to an automatic process or also requires controlled attention. de la Rosa et al. (2012) investigated this issue by using a dual-task paradigm. In a single task, participants performed a simple RT detection task in which participants were instructed to respond to an auditory target preceded by a regular or an irregular auditory rhythm. In the dual task, participants simultaneously performed a working memory (WM) task within visual modality. The results showed that participants could predict the target onset time based on the rhythm, and this effect did not suffer from dual-task interference. They suggested rhythmic temporal expectation did not demand controlled attentional resources, and demonstrated an automatic nature underling rhythm based temporal expectation (de la Rosa et al., 2012). However, their experiment used a visual WM task based on auditory rhythms, the two simultaneous tasks involved distinct sensory modalities. According to the multi-resources model (Wickens, 2008), when the stimulus processing in two simultaneous tasks share the same sensory modality, the interference is the greatest. Therefore, in their study, whether the finding of temporal expectation effect survived dual-task interference was caused by the rhythms and the stimuli to be remembered with the distinct modality remains to be further addressed. Thus, whether rhythm-based temporal expectations rely upon automatic processing independent of controlled processing has not been clearly established.

Tempo refers to the rate (or pace) of an isochronous sequence of a stimulus (namely, how fast or slow it is) and is commonly expressed as the time interval between successive stimuli in the sequence. Most previous research on rhythm-based temporal expectations has focused only on the intervals of <1 s (subsecond range) (Lange, 2010; Sanabria et al., 2011; de la Rosa et al., 2012; Miller et al., 2012; Sanabria and Correa, 2013; Breska and Deouell, 2016; Jones et al., 2017). Several studies have proposed distinct mechanisms during the measurement of durations at the subsecond and suprasecond ranges. They suggested that time measurements at the subsecond range are automatic while those at the suprasecond range require the involvement of cognitive control (Rammsayer, 1999; Lewis and Miall, 2003a,b; Rammsayer and Ulrich, 2011). Moreover, in the above studies, participants were generally exposed to duration discrimination tasks, in which they were instructed to provide an overt estimate of stimulus duration or intervals, whether one duration is shorter or longer than another. However, when participants were exposed to a temporal expectation task, in which they were simply asked to respond to the target as rapidly and accurately as possible, and they were unconscious that they were processing time, whether the temporal expectation induced by a rhythm of subsecond and suprasecond interonset intervals (IOI) also involves different neural systems has rarely been compared directly. In other words, whether the mechanisms underlying rhythmic temporal expectation may change with the tempo is not clearly established.

To summarize, two main issues inspired our present study. First, whether temporal expectation driven by rhythms is dependent on automatic processing and does not require the involvement of controlled mechanisms, even if the stimuli in the simultaneously performed dual tasks share the same modality. To clarify this issue, we adopted a dual-task paradigm, in which the stimuli in the two concurrent tasks with the same visual modalities. To test whether the temporal expectation effect induced by rhythm would resist the interference from the double task share the same modality. As in a classic dual-task paradigm, two tasks, namely, the primary and the secondary tasks, are executed concurrently. In our dual-task experiment, we used a simple RT detection task as the primary task to measure the temporal expectation effect. Participants were required to respond to a visual target presented after a visual stimuli sequence (regular vs. irregular). A visual WM task was performed as the secondary task, in which participants required to remember a set of six different letters in each trial. The logic of our design was based on the criterion that if temporal expectation driven by rhythm involves controlled processing, then performance on the primary simple RT detection task should be impaired by the WM secondary task because the primary and secondary tasks compete for common limited resources. In contrast, if rhythm-based temporal expectations rely on automatic processing and are independent of resources of executive control, then the temporal expectation effect would not suffer from dual-task interference (Logan, 1978, 1979; Posner and Snyder, 2004).

Second, whether the mechanisms underlying temporal expectation driven by rhythm may change with the tempo. Specifically, whether different neural systems existent for the rhythm-based temporal expectation with subsecond and suprasecond IOI. To clarify this issue, in the present study, we included two IOI, 500 ms for the fast tempo and 3,500 ms for the slow tempo. That way, we were able to investigate the effect of tempo on the nature of the processes involved in rhythmic temporal expectation. To verify the finding in study of de la Rosa et al. (2012), and combined the IOI frequently used in the previous studies related to rhythmic temporal expectation (Lange, 2009, 2010; Sanabria and Correa, 2013; Ren et al., 2019; Xu et al., 2020), we chose 500 ms IOI for the fast tempo. Moreover, the previous studies have shown that the limit of IOI that participants can synchronize was up to 3,500 ms (Repp, 2007). Thus, we chose 3,500 ms as the slow tempo. We hypothesized that under the 500 ms fast tempo condition, the temporal expectation effect would resist the dual-task interference. In contrast, under the 3,500 ms slow tempo condition, the temporal expectation effect would be reduced by the simultaneous WM task.

## Materials and Methods

### Participants

In this study, 34 healthy, right-handed students (mean age, 21.4 years; range, 20–22 years; 31 men and 3 women) from Okayama University took part as volunteers. All participants had normal or corrected to normal vision and without psychiatric disorders. Participants reported that they had not received professional music training and played a musical instrument 3 years before the experiment. The study was based on approval from the institutional ethics committee of Okayama University, and all participants gave a written informed consent prior to their enrollment. Participants were randomly assigned to the single-task (17 participants) and dual-task (17 participants) conditions. Random assignment was ensured by using software known as the “Research Randomizer,” which is available online^1^ (Urbaniak and Plous, 2011). One participant in each of the two tasks was excluded because of the problems during data collection.

### Apparatus and Stimuli

**FIGURE 1:**
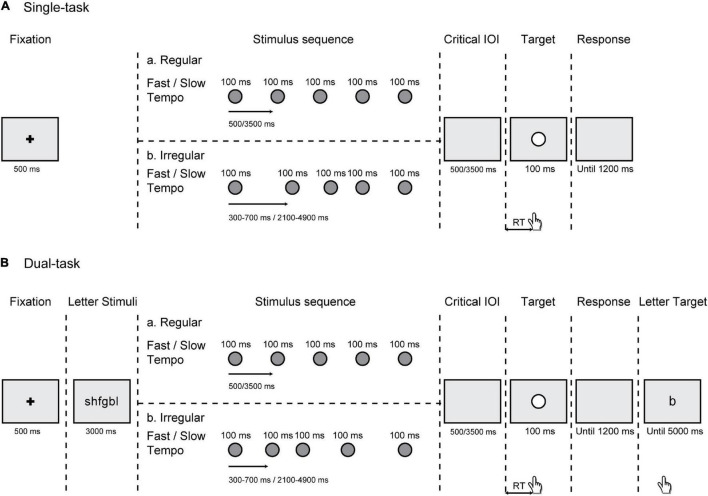
Schematic representation of events in a trial. **(A)** In the single-task, participants responded to a white circle target preceded by either a regular or an irregular rhythm under a fast (500 ms) or slow (3,500 ms) tempo. **(B)** In the dual-task, participants simultaneously performed a working memory (WM) task, in which they had to remember a group of six letters that appeared for 3,000 ms preceding the stimulus sequence.

We used E-prime software (Schneider et al., 2002) for stimulus appearance and participant RT recording. All stimuli were displayed on the center of a 27-inch computer monitor at a resolution of 1,280 × 720 pixels, with a gray background (RGB = 180, 180, 180). In both the single- and dual-task, each trial began with a fixation point (black “+,” 0.6° × 0.6°) presented for 500 ms. The stimulus sequence consisted of five or six successive circles (uniform probability) with a duration of 100 ms each. All the stimuli in the sequence were gray solid circles (diameter = 1.2°; RGB = 100, 100, 100). Different numbers of gray circles were designed to prevent the target from being completely predictable. This sequence was regular or irregular according to a uniform probability distribution. In the regular sequence, the IOI of the gray circles were 500 ms (fast tempo) or 3,500 ms (slow tempo), which changed from trial to trial. In the irregular sequence, the IOI could be 300, 400, 500, 600, and 700 ms (fast tempo) or 2,100, 2,800, 3,500, 4,200, and 4,900 ms (slow tempo). The order of these five intervals was randomly presented across trials. The target was a white circle target (diameter = 1.2°) with a duration of 100 ms. In addition, in the dual-task conditions, a group of six different letters randomly generated in the consonants of alphabet was presented preceding the stimulus sequence. And at the end of each trial, after the response to the white circle target, a response letter for the memory task was displayed on the monitor. All letters were presented in “Arial” font and 30-pixel font size (Figure 1).

### Procedure and Task

Participants were seated comfortably on a chair in a quiet, dimly illuminated room, with a viewing distance of 60 cm from the center of the screen and a chin rest held their head positions. For the single- and dual-task, participants were provided with the verbal and written instructions. Each task contained one practice block and 10 experimental blocks, each consisting of 30 trials comprising 150 fast tempo trials and 150 slow tempo trials (75 trials for regular and irregular conditions each). A schematic of both tasks is provided in Figure 1.

In the single-task, at the beginning of each trial, a fixation point was presented for 500 ms. Then, a regular or irregular sequence was randomly presented. Following the sequence, a white circle target appeared after 500 ms (fast tempo) or 3,500 ms (slow tempo). Participants were instructed, when the target was presented to press the “↑” key button as soon as possible. In 20% of trials (60 trials), the target was not presented (catch trials). We designed catch trials to avoid the “hazard function” effect. In the case that the stimulus has not yet occurred, expectations were generated by the conditional probability that the stimulus would appear (Correa et al., 2006). The maximum allowable response time was 1,200 ms. The total duration of each trial (except RT) was 3.35 s for fast tempo, and 19.85 s for slow tempo trial, both regular and irregular trials had identical duration.

Under the dual-task, the process was nearly the same as that described for the single-task, except that the subjects concurrently performed a WM task. Before presenting the regular or irregular sequence, a group of six letters to be remembered would appear for 3,000 ms. At the end of each trial, after the response to the white circle target, a response letter for the memory task was displayed on the monitor. If the response letter was contained in the group of six letters, participants were required to press the “←” key button, and if the response letter was not included in the preceded group of letters, press the “→” key. A maximum of 5,000 ms was allowed for the response. After the participants responded, a word “correct” or “incorrect” in blue or red, respectively, was presented for 500 ms, providing feedback on accuracy of memory.

### Design and Data Analysis

The experiment consisted of a 2 (task: single/dual) × 2 (rhythm: regular/irregular) × 2 (tempo: fast/slow) mixed factor design. The task was between-participants variables. Rhythm and tempo were within-participants variables. Regular and irregular conditions and fast and slow tempo conditions randomly appeared in each trial, and the probability of occurrence was the same.

The RT was defined as the time duration between the initiation of target and the first observable response in both tasks. Data from practice blocks and catch trials were excluded from the analyses. The anticipatory responses (a response that appeared before the target being presented), omission errors (participants miss to press the key in response to the target), and RTs below 150 ms or above 1,200 ms were eliminated from the RT analysis. Overall mean remaining trials of participants were 116 (4.5 SD) fast and 118 (2.9 SD) slow tempo trials in a single-task condition, and 117 (2.8 SD) fast and 119 (1.4 SD) slow tempo trials in the dual-task condition. The remaining correct mean RTs were analyzed using repeated-measures analysis of variance (ANOVA). Overall mean remaining correct trials of participants were 102 (8.4 SD) fast and 105 (4.6 SD) slow tempo trials in the dual-task condition. In a subsequent analysis, we checked the temporal expectation effect (difference of the RT in regular and irregular trials) instead of the mean RTs, analyzed using a repeated-measures ANOVA with a 2 (task: single/dual) × 2 (tempo: fast/slow) design.

In addition, the mixed-effects linear regression analyses (Pinheiro and Bates, 2000; Baayen et al., 2008) were conducted to re-examined the data, using the *lmer* function from in *lme4* package (Bates et al., 2015) for R (vision 4.0.2). The models were built for temporal expectation effect. The fixed-effect predictors included: Task (single and dual) and Tempo (fast and slow) as the categorical predictors; Block number as the continuous predictor, and their interactions were included in all the models. Block numbers were centered to minimize collinearity. For Task and Tempo, simple coding (single-task: −0.5; dual-task: 0.5; fast tempo: −0.5; slow tempo: 0.5) was used.

## Results

Overall mean accuracy of participants on the letter memory test was 87.5% (7% SD) in the dual-task condition. Exactly, 86.9% for the fast (irregular 86.2% and regular 87.7%) and 88.1% for the slow (irregular 87.8% and regular 88.4%) tempo and was not different between conditions (all *p* > 0.05). The RT was only analyzed using accurate responses in the memory test to ensure that subjects were absolutely involved in the dual-task state.

**FIGURE 2:**
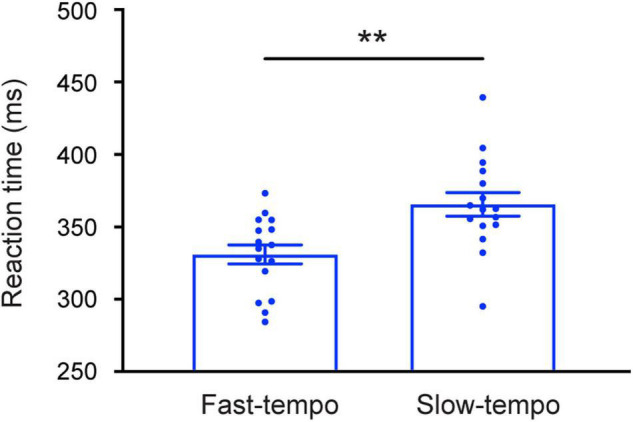
Mean reaction times (RTs) were faster following the fast rhythm than slow rhythm. Error bars represent the SEM. **p < 0.01.

Detailed mean RTs for each condition are presented in Table 1. The 2 _Task_ (single and dual) × 2 _Tempo_ (fast and slow) × 2 _Rhythm_ (regular and irregular) ANOVA revealed that the main effect of task was significant [*F*_(1,30)_ = 8.387; *p* = 0.007; η^2^ = 0.218], showed the RTs for the single-task (334.54 ms) were faster than for the dual-task (359.49 ms). A significant main effect of Tempo [*F*_(1,30)_ = 8.720; *p* = 0.006; η^2^ = 0.225 ms) was also revealed, showing that the RTs were significantly shorter in the fast tempo condition (329.68 ms) than in the slow tempo condition (364.35 ms) (Figure 2). In addition, there was a significant main effect of rhythm [*F*_(1,30)_ = 60.137; *p* < 0.001; η^2^ = 0.667], which showed that the RTs for regular trials were faster than for irregular trials.

**TABLE 1:** Mean reaction times (RTs) (ms) for each Tempo (fast and slow), Rhythm (regular and irregular), and Task condition (single-task and dual-task).

	Single-task		Dual-task	
	**Fast**	**Slow**	**Fast**	**Slow**

Regular	298 (7)	316 (9)	324 (8)	374 (14)
Irregular	338 (10)	387 (15)	360 (14)	380 (14)

*Values in parentheses are SEM.*

**FIGURE 3:**
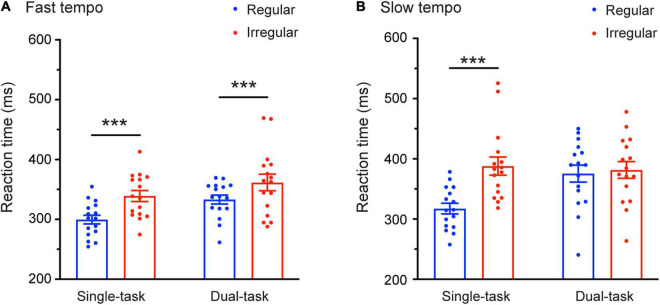
Mean reaction times (RTs) as a function of the Rhythm (regular and irregular) and Task condition (single-task and dual-task) for the fast **(A)** and slow **(B)** tempo. Error bars represent the SEM. ***p ≤ 0.001.

There was a significant interaction between task and rhythm [*F*_(1,30)_ = 11.81, *p* = 0.002, η^2^ = 0.282]. Most relevant to our prediction was the finding of a significant three-way interaction: Task × Tempo × Rhythm [*F*_(1,30)_ = 6.968, *p* = 0.013, η^2^ = 0.188]. Further analysis of this interaction revealed that the Task × Rhythm interaction was significant only under the slow tempo condition [*F*_(1,30)_ = 14.761, *p* = 0.001, η^2^ = 0.33] but not under the fast tempo condition [*F*_(1,30)_ = 0.049, *p* = 0.826, η^2^ = 0.002]. Follow-up pairwise comparisons (Bonferroni corrected) showed that during the slow tempo condition, the mean RTs for the regular trials (316.18 ms) were significantly faster than for the irregular trials (386.69 ms) in the single-task condition (*p* < 0.001), whereas under the dual-task condition, no significant difference was found between the regular and irregular trials (*p* = 0.618). During the fast tempo condition, the mean RTs for the regular condition were significantly faster than for the irregular conditions in both the single- (*p* < 0.001) and dual-task (*p* = 0.001) (Figure 3).

**FIGURE 4:**
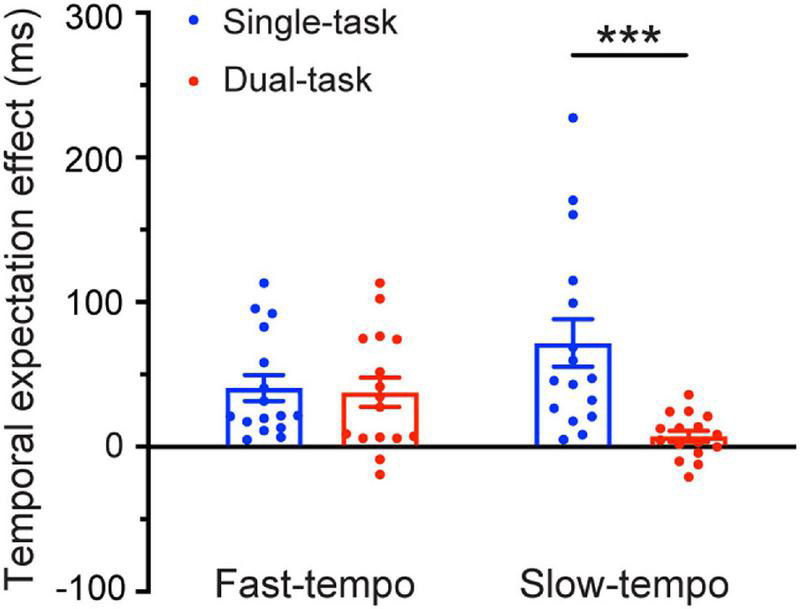
Temporal expectation effects [irregular reaction time (RT) minus the regular RT] as a function of the Tempo (fast and slow) and Task condition (single-task and dual-task). Error bars represent the SEM. ***p ≤ 0.001.

Regarding the temporal expectation effect (difference of the RT in regular and irregular trials). The 2 _Task_ (single, dual) × 2 _Tempo_ (fast, slow) ANOVAs revealed a significant main effect of the task [*F*_(1,30)_ = 11.81, *p* = 0.002, η^2^ = 0.282]. The temporal expectation effect for single-task (54.97 ms) was larger than for the dual-task (21.21 ms). The interaction between the task and the tempo was significant [*F*_(1,30)_ = 6.968, *p* = 0.013, η^2^ = 0.188]. Follow-up pairwise comparisons (Bonferroni corrected) showed that the temporal expectation effect for the single-task (70.51 ms) was significantly larger than for the dual-task (5.98 ms) during the slow tempo condition (*p* = 0.001). In contrast, during the fast tempo condition, there was no significant difference between the single-task and dual-task (*p* = 0.826) (Figure 4).

**FIGURE 5:**
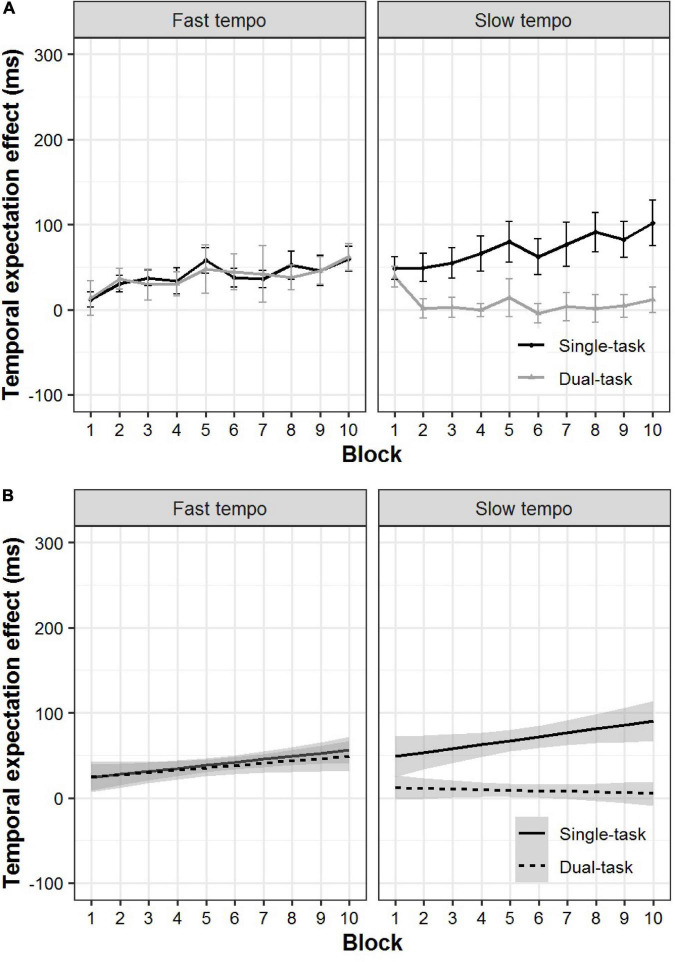
Actual plots **(A)** and model prediction plots **(B)** for the temporal expectation effect of the single- and dual-task for the fast and slow tempo conditions as a function of block. The 95% CIs are shown in gray shading.

We re-examined the data in terms of the temporal expectation effect using the mixed-effects linear regression analyses. We still observed a significant main effect of task (β = 32.64, *p* < 0.001). The most relevant finding to our prediction was that we still found a significant interaction between task and tempo (β = −62.59, *p* < 0.001). This result showed that the temporal expectation effect for a single-task was significantly larger than for the dual-task during the slow tempo condition (*p* < 0.001). In contrast, during the fast tempo condition, there was no significant difference between the single-task and dual-task (*p* = 0.86) (Figure 5).

## Discussion

The goal of the present experiment was to explore whether temporal expectation driven by rhythms is dependent on automatic processing or requires the involvement of controlled mechanisms and whether the mechanisms underlying temporal expectation driven by rhythm may change with the tempo. To clarify this issue, we used a dual-task paradigm to test the underlying mechanisms of temporal expectation driven by fast and slow rhythms. We hypothesized automatic mechanisms underlying fast tempo guided temporal expectation. However, controlled processing was involved in slow tempo guided temporal expectation. The results showed that during the fast tempo condition, the RTs for the regular conditions were faster than for the irregular conditions in both the single- and dual-task, and the temporal expectation effect was not affected by extra processing demands. However, during the slow tempo condition, faster RTs for the regular trials than for the irregular trials were only found under the single-task, and the temporal expectation effect was attenuated by the secondary task.

Faster RTs for a single-task compared with the dual-task confirmed that our manipulation of the WM task was effective. The main effect of rhythm demonstrated that participants could use rhythms to create temporal preparation, which captured the temporal expectation effect with faster RTs for the regular trials than for irregular trials. Most relevant, the temporal expectation effect survived to dual-task interference under fast tempo conditions. Such findings are consistent with the study of de la Rosa et al. (2012), who used a visual WM task based on auditory rhythms, with two simultaneous tasks involved distinct sensory modalities. In our present study, we used a visual WM task based on visual rhythms. In this way, we went a step further, combined single- and dual-tasks that shared the same sensory modality, thereby increasing the degree of interference. Our results confirmed and added to the existing literature by showing that rhythm-based temporal expectation is created in an automatic, unintentional manner and is not prone to interference by the controlled, intentional processes. In addition, we further propose that the automatic mechanisms underlying rhythmic temporal expectation are limited by the time scale.

Compared with the fast tempo condition, during the slow tempo condition, the temporal expectation effect was impaired by the dual-task requirements. The RTs of participants were indeed shorter for regular conditions than for irregular conditions only under the single-task, and no significant difference was found between the regular and irregular conditions under the dual-task. This result indicated the effect of tempo on the mechanisms underlying rhythmic temporal expectation. Compared with the faster tempo, the slower tempo might involve more controlled processing. The pattern of results can be partly interpreted by the fact that if the rhythm is too slow, then, the rhythmic organization may tend to be weakened, and then diminished the ability to entrain the organization into alignment with external periodic stimulation to predict the future events. In this case, the brain may likely draw support from a memory-based method to complement the form of temporal expectations. The memory-based way has been related to the interval models, in which three independent processing components: estimating the interval, storing interval information as a reference, and measuring the ongoing interval relative to that of a remembered interval, when the ongoing interval reaches the interval standard, then the presently timed interval is expected to end (Gibbon, 1977; Church et al., 1983). Because the secondary task (WM task) in the present study requires WM maintenance and rehearsal. When the temporal expectation draws support from the memory-based that requires controlled processing, it will compete for common limited resources with WM tasks. As a result, temporal expectation effects were reduced by the WM task.

In line with our previous research (Ren et al., 2019; Xu et al., 2020), the present study showed faster RTs for the fast tempo condition than for the slow tempo condition. This finding can be explained by the same conjecture applied to the case of the slow tempo condition, in which temporal expectation driven by rhythm engage controlled processing. Our previous studies have suggested that exogenous temporal expectations had a faster response and provided a more precise attentional focus in time compared with endogenous temporal expectation. The current results have once again verified our conjecture. However, even if we found that temporal expectation driven by rhythms required attentional control, as the tempo slows down, whether automatic processing is completely replaced by controlled processing or both coexist, still needs to be further confirmed. In addition, in the current study, we chose only two IOI (500 ms for fast tempo and 3,500 ms for slow tempo) to investigate the effect of tempo on mechanisms underlying rhythmic temporal expectation. Necessarily, a future challenge will be to set up more IOI to systematically track how rhythmic temporal expectation evolved over time.

In addition, one question should be taken into consideration. In the dual-task condition, participants were presented with letters to memorize before presenting the visual sequence (rhythmic temporal cue). In contrast, in a single-task condition, nothing was presented. The dual-task differed in terms of visual input and cognitive load. Therefore, it could be argued that the reduced temporal expectation effect was induced by the cognitive load or just because the visual input differs. In light of the study by Capizzi et al. (2012), they utilized a dual-task paradigm that proved more controlled processing in temporal expectation driven by symbolic temporal cues. Indeed, their single-task and dual-task condition both included the same visual input (three colored stars). In single-task conditions, participants were instructed to ignore it, while in dual-task conditions, participants needed to remember the final count of each color. Their results reported a significant temporal expectation effect for single-task conditions, although with a visual input before the temporal cue (Capizzi et al., 2012). Thus, we conjectured that if the extra visual input before the presentation of the temporal cue would induce the temporal expectation effect to disappear, as found in our slow tempo results. Then, it ran counter to observe the significant temporal expectation effect reported by Capizzi et al. (2012). Therefore, we reasoned that the visual input differs might not be the main reason for impaired temporal expectation effect of our slow tempo condition. However, future research is required to examine the damage of the temporal expectation effect is due to visual input or the cognitive load.

Another argument might be the between-subject design with the task (single and dual) as the between-participants factors. It could be disputed that our finding of temporal expectation effect impaired under dual-task interference during the slow tempo condition was partly caused by differences between the groups, e.g., different sensitivity to rhythmic cues. However, first of all, all participants reported that they had not received professional music training and played a musical instrument 3 years before the experiment. And we randomly assigned subjects. Moreover, if the impaired temporal expectation effect during slow tempo condition was caused by the differential sensitivity to rhythmic cues between groups. Then, during the fast tempo condition, we should also find a reduced temporal expectation effect. Whereas, our results contrarily showed that the temporal expectation effect under fast tempo was not affected by the secondary task. Therefore, we conjecture that our finding of temporal expectation effect impaired under dual-task interference during the slow tempo condition might not be caused by differences between the groups. Inevitably, a future study would need to examine the current finding by within-subject design further.

## Conclusion

In conclusion, the results of the present study demonstrated that the temporal expectation effect triggered by rhythms did not suffer from dual-task interference under fast tempo conditions but was impaired under slow tempo conditions. These results indicated that rhythm-based temporal expectations were dependent on automatic processing only on limited time scales. Compared with the automatic mechanisms underlying faster tempo, the rhythmic temporal expectation induced by slower tempo might involve more controlled processing. These outcomes revealed that the mechanism underlying temporal expectation driven by rhythms may not be immutable and will change under the influence of tempo.

## Data Availability Statement

The raw data supporting the conclusions of this article will be made available by the authors, without undue reservation.

## Ethics Statement

The studies involving human participants were reviewed and approved by the Okayama University. The patients/participants provided their written informed consent to participate in this study.

## Author Contributions

ZX and YR conceived and designed the experiments. YM collected the data. ZX analyzed the data, wrote the draft manuscript, and received comments from QW and SL. All authors contributed to the article and approved the submitted version.

## Conflict of Interest

The authors declare that the research was conducted in the absence of any commercial or financial relationships that could be construed as a potential conflict of interest.

## Publisher’s Note

All claims expressed in this article are solely those of the authors and do not necessarily represent those of their affiliated organizations, or those of the publisher, the editors and the reviewers. Any product that may be evaluated in this article, or claim that may be made by its manufacturer, is not guaranteed or endorsed by the publisher.

## Footnotes

1
www.randomizer.org
